# Surgical outcomes of the endoscopic transsphenoidal route to pituitary tumours in paediatric patients >10 years of age: 5 years of experience at a single institute

**DOI:** 10.1136/archdischild-2015-308365

**Published:** 2015-05-25

**Authors:** Rucai Zhan, Guangming Xu, Timothy M Wiebe, Xingang Li

**Affiliations:** 1 Department of Neurosurgery, Qilu Hospital of Shandong University, Brain Science Research Institute, Shandong University, Jinan, Shandong, China; 2 Department of Neurosurgery, Shandong Provincial Hospital Affiliated to Shandong University, Jinan, Shandong, China; 3 Private practice, Bakersfield, California, USA

**Keywords:** Neurosurgery, Oncology, Paediatric Surgery

## Abstract

**Objective:**

To evaluate the safety and effectiveness of the endoscopic endonasal transsphenoidal approach (EETA) for the management of pituitary adenomas in paediatric patients >10 years of age.

**Methods:**

A retrospective chart review was performed to identify 56 paediatric patients between 10 and 18 years of age who underwent an endonasal endoscopic transsphenoidal approach for the resection of a pituitary adenoma during the last 5 years. The age, sex, symptoms, tumour size, extent of tumour resection, clinical outcome and surgical complications of patients were reviewed.

**Results:**

Total resection was achieved in 49 (87.5%) cases, subtotal resection was achieved in 7 (12.5%) cases and no patient had a partial or insufficient resection. Of the 35 patients who experienced preoperative deterioration of vision, 33 (94.2%) achieved visual remission with rates of 34.2% and 60% for normalisation and improvement, respectively. Endocrinological normalisation was achieved in 13 (31.7%) of 41 patients who had preoperative hyperhormonal levels; hormone levels decreased in 25 (61.0%) patients, and 3 (7.3%) patients had no change in hormone level. Two (3.5%) patients incurred postoperative cerebrospinal fluid leakage, which was resolved after lumbar drainage. Four (7.1%) patients developed hypopituitarism, which required hormone therapy. Post-surgery, five (8.9%) patients incurred transient diabetes insipidus (DI), of which one (1.7%) patient developed persistent DI and was administered Minirin. Meningitis occurred in one (1.7%) patient who was cured by the administration of a third-generation antibiotic. There were no cases of intracranial haematoma, reoperation or death.

**Conclusions:**

EETA allows neurosurgeons to safely and effectively remove paediatric pituitary adenomas with low morbidity and mortality.

**What is already known on this topic?**

- Endoscopic transsphenoidal surgery is a novel and minimally invasive approach for pituitary adenomas.
- This technique may be more challenging in paediatric patients due to incomplete development of the nasal and sphenoid sinuses.

**What this study adds?**

- The surgical outcomes of endoscopic transsphenoidal resection for pituitary adenomas in older paediatric patients were excellent and comparable to those of adults.
- The postsurgical complications of the endoscopic transsphenoidal approach exhibited a low incidence and were curable.
- Endoscopic transsphenoidal surgery can be performed safely in paediatric patients older than 10 years of age with low morbidity and mortality.

## Introduction

Although endoscopic transsphenoidal surgery has become a mainstay for the management of tumours of the sellar region in many centres since Apuzzo *et al*^1^ reported their experience with an angled endoscope for transsphenoidal surgery in 1977, this procedure is more challenging in paediatric patients due to anatomical limitations of the nasal and sphenoid sinuses. However, these limitations do not apply to all age groups of paediatric patients. In the second decade of the childhood, the endoscopic transsphenoidal approach may become available for resection of a sellar tumour. According to anatomical studies of the nasal and sphenoidal sinuses,^2^ ^3^ the sphenoidal sinus is well developed in most of the children >10 years of age, although the sphenoidal separation is not well formed in some children younger than 14 years. Therefore, we postulated that the endoscopic transsphenoidal approach may benefit paediatric patients >10 years of age, and we reported our early experience of endoscopic resection for pituitary tumours in patients of this age group.

## Methods

### Experimental design

We retrospectively reviewed the hospital records of 550 patients admitted to Qilu Hospital of Shandong University between July 2009 and June 2014, and 65 patients between 10 and 18 years of age at the time of diagnosis were identified. Nine patients who underwent a bifrontal transbasal approach due to a conchal type of sphenoid sinus were excluded from this study; the remaining 56 patients underwent endonasal endoscopic transsphenoidal resection of a pituitary tumour. The records of these patients were reviewed, and the age, sex, presentation, tumour size, extent of tumour resection, clinical outcome and surgical complications of the patients were recorded.

### Preoperative examinations

**Figure 1:**
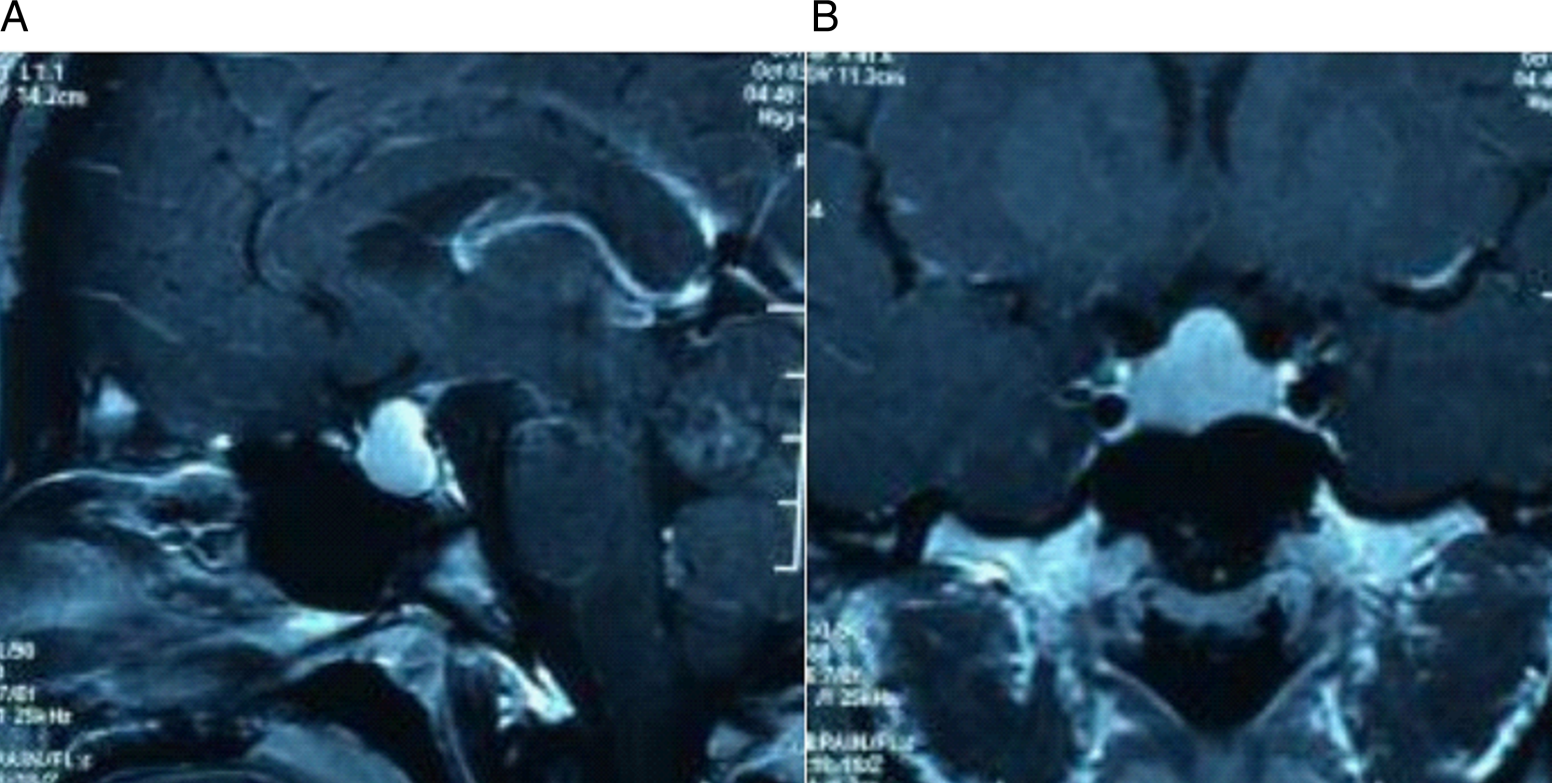
Preoperative MRI. Sagittal (A) and coronal (B) enhanced T1-weighted MRI of a 16-year-old male that presented with gigantism and headache. The images show the tumour extending into the suprasellar cistern.

All patients underwent a detailed preoperative assessment within 24 h of hospitalisation. In addition to an ECG and standard blood examinations, including glycaemic evaluation, blood cell counts and serum and urinary Na^+^ levels, a hormonal evaluation was performed to evaluate the pituitary function and the levels of prolactin (PRL), growth hormone (GH) and adrenocorticotropic hormone (ACTH). Visual assessments were performed, including visual reflex, acuity and field evaluations, to determine the scope of visual deterioration, and the findings were compared with the results after surgery. MRI at 3.0 T was conducted in 56 patients to assess the development of the nasal cavity, pneumatisation of the sphenoid sinus and the characteristics of the pituitary adenoma, such as the location, extent and relationship to surrounding structures (figure 1). In addition, histopathological examinations were performed on all excised specimens.

### Endoscopic technique

**Figure 2:**
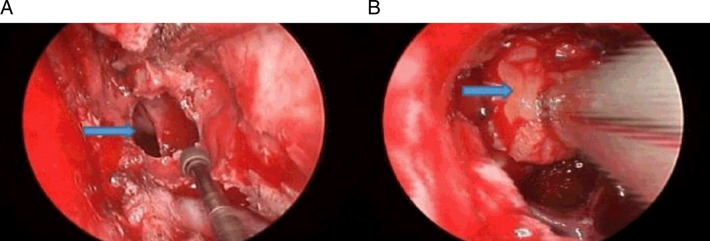
Intraoperative endoscopic views. The patient underwent surgery via endoscopic endonasal transsphenoidal approach after admission. According to the intraoperative endoscopic views, the sphenoid sinus was completely pneumatised (arrowhead) (A), and the tumour was removed by the microsurgical instrument (arrowhead) (B).

All patients received intraoperative prophylaxis with third-generation cephalosporin. Usually, the right lateral thigh was prepared for harvesting the fascia lata or fat used to repair the skull base if needed. The first step in the procedure consisted of introducing a 0° rigid endoscope, 4 mm in diameter and 18 cm in length, into the right nostril. At the beginning of the operation, if a cerebrospinal fluid (CSF) leakage was anticipated, such as in case of macroadenoma, a vascularised pedicled nasoseptal flap (PNSF) was harvested and rotated into the posterior nasopharynx for later use for reconstruction of skull base. The middle turbinate was located and pushed laterally, and the sphenoid ostium—a key anatomical landmark—was located without difficulty. Then, the posterior nasal septum was dissected, and a wide opening was made in the anterior wall of the sphenoid sinus (figure 2). After removal of the sphenoid sinus septations, the sellar floor was opened with a high-speed microdrill.

The tumour was explored and removed with a curette or with suction after the dura of sellar floor was opened cruciately. Suprasellar tumours typically dropped down into surgical view due to CSF pulsations, and they were easily resected. After the operation was finished, a 30° endoscope was used to detect and remove the residual tumour. The surgical cavity was filled with Gelfoam. In cases in which an intraoperative CSF leak was confirmed or suspected, an autologous fat or fascia lata graft was placed within the dural defect as an inlay graft, and a synthetic dural graft was applied as an overlay graft. The PNSF was then rotated and positioned over the defect at the skull base, and fibrin glue was applied to the synthetic dura. The sphenoid sinus was routinely filled with Gelfoam, and the nasal cavity was packed with pledgets.

### Postoperative management

**Figure 3:**
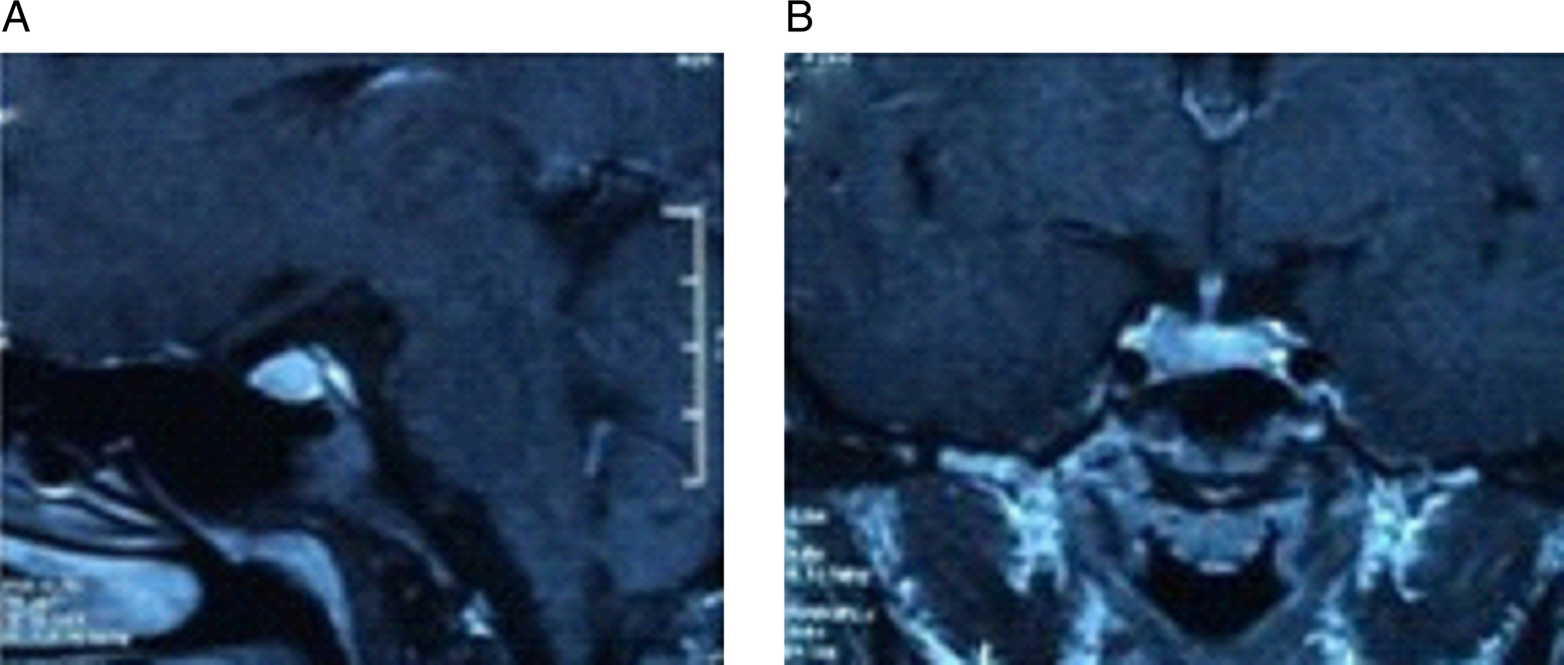
Postoperative MRI after 1 year of follow-up. Sagittal (A) and coronal (B) enhanced T1-weighted MRI after 1 year of follow-up. The postoperative images show complete absence of the tumour.

The third-generation cephalosporin was continued for 3–7 days. Fluid intake, urine output, serum electrolytes and cortisol levels were monitored. Hormone replacement therapy was administered in cases in which postoperative hormone insufficiency occurred. MRI of the sella was performed postoperatively within 1–3 days and at 3 months to evaluate the extent of tumour resection (total resection (TR): no evidence of residual tumour; sub-TR: residual tumour <20%; partial resection: residual tumour <50%; and insufficient resection: residual tumour >50%)^4^ (figure 3). Nasal packing was generally removed endoscopically within 1–3 days after surgery. Patients were instructed to maintain bed-rest with their head in a slightly elevated position and to avoid any activity that might raise intracranial pressure, such as straining or nose blowing. Lumbar drainage was performed on a patient who had persistent postoperative CSF leakage. Transient postoperative diabetes insipidus (DI) was managed by subcutaneous injection of Hypophysin, and persistent DI was treated with daily administration of Minirin, a desmopressin. We did not use controlled release vasopressin tannate because its effects may be difficult to control, and it is an inconvenient method of treatment for patients.

## Results

Fifty-six patients were included in this cohort, which consisted of 36 (64.2%) males and 20 (35.8%) females; 5 patients were 10–14 years old, and 51 patients were 15–18 years old (a total mean of 16.6 years). The follow-up period ranged from 6 to 128 months (mean 52 months). The most common initial complaints were deterioration of vision (35/56, 62.5%) and headache (25/56, 44.6%), followed by accelerated development (20/56, 35.7%), amenorrhoea and galactorrhoea (16/56 28.6%). Five (8.9%) of the patients had symptoms of Cushing's disease. Fourteen patients had microadenomas, and the remaining 42 (75%) patients had an adenoma and a varied extent of suprasellar and cavernous sinus tumours (table 1).

**Table 1:** Summary of the clinical characteristics of 56 patients

	No	Rate (%)
Sex		
Male	36	64.2
Female	20	35.8
Age group		
10–14	5	8.9
15–18	51	91.1
Presentations		
Visual loss	35	62.5
Accelerated development	20	35.7
Amenorrhoea, galactorrhoea	16	28.6
Cushing's disease	5	8.9
Headache	25	44.6
Extent of adenoma		
Intrasellar	14	25
Suprasellar extension	21	37.5
Cavernous sinus extension	9	16.1
Suprasellar and cavernous sinus extension	12	21.4

TR was achieved in 49 (87.5%) cases, sub-TR was achieved in 7 (12.5%) cases and no patient had a partial or insufficient resection. Also, 33 (94.2%) of 35 patients who experienced preoperative deterioration of vision achieved visual remission with rates of 34.2% and 60% for normalisation and improvement, respectively. Endocrine function was thought as normalisation if hormone level postoperatively decreased to standard reference range of endocrinology, such as PRL 3.34–26.72 ng/mL, GH 0.126–9.88 ng/mL and ACTH 7.2–63.3 pg/mL. After surgery, endocrine function was normalised in 13 (31.7%) of 41 patients who had preoperative hyperhormonal levels, hormone levels were decreased in 25 (61.0%) patients and 3 (7.3%) patients had no change in hormone levels (table 2).

**Table 2:** Surgical outcomes

	No	Rate (%)
Extent of resection		
Total resection	49	87.5
Subtotal resection	7	12.5
Pathological type		
Secreting	41	73.2
PRL	15	26.8
GH	20	35.7
ACTH	6	10.7
Non-secreting	15	26.8
Endocrinological recovery		
Normalised	13/41	31.7
Improved	25/41	61.0
No changed	3/41	7.3
Visual recovery		
Normalised	12/35	34.2
Improved	21/35	60
No changed	2/35	5.8

Two (3.5%) patients incurred postoperative CSF leakage, which was resolved after lumbar drainage. Four (7.1%) patients developed hypopituitarism, which required hormone therapy. Post-surgery, five (8.9%) patients incurred transient DI, of which one (1.7%) patient developed persistent DI, which was treated with Minirin. Meningitis occurred in one (1.7%) patient who was cured by a third-generation cephalosporin. There were no cases of intracranial haematoma, reoperation or death (table 3).

**Table 3:** Surgical complications

	No	Rate (%)
Cerebrospinal fluid leakage	2	3.5
Transient diabetes insipidus	5	8.9
Persistent diabetes insipidus	1	1.7
Hypopituitarism	4	7.1
Meningitis	1	1.7
Reoperation	0	0
Intracranial haematoma	0	0
Death	0	0

## Discussion

Paediatric pituitary adenomas are rare and account for 2.1–6% of all pituitary tumours managed by a surgical approach.^5^ Although there is a low incidence rate of pituitary adenoma in childhood and adolescence, the tumour may significantly affect the development and growth of the patient during puberty. Early detection and surgical treatment may produce a better outcome.^5^ Although transsphenoidal surgery for the resection of sellar tumours is common, there is currently little information in the literature regarding the paediatric endoscopic transsphenoidal approach; one reason may be associated with the definition of paediatric patients. The reported cut-off age varies from 16 to 20 years,^6–8^ and accordingly, the incidence of pituitary adenoma ascribed to paediatric populations is affected by how one defines ‘paediatric’. For instance, a pituitary adenoma is relatively rare in childhood, and the incidence increases during adolescence, which extends through 19 years of age.^9^ Another factor is that endoscopic surgery in paediatric patients is a challenging technique due to anatomical limitations in paediatric patients and surgical instruments. However, progression in the anatomical study of the skull base and nasal cavity has made endoscopic surgery available in selected paediatric patients. Tatreau *et al*^3^ reported a radio-anatomic cross-sectional survey of 50 paediatric patients and indicated that sphenoid pneumatisation is not an anatomical limitation in paediatric patients older than 10 years of age. In the present study, only 9 (13.8%) of 65 patients had a conchal sphenoid sinus, the remaining 56 paediatric patients showed good sphenoid pneumatisation on radiographic images. The rate of sphenoid pneumatisation was 86.2%, which supported the findings of Tatreau. Patients undergoing puberty are thought have a high incidence of pituitary adenomas. Tetsuro *et al*^10^ reported that in a series of paediatric adenomas the majority of patients were between 14 and 18 years of age, and most had secreting macroadenomas. We found that the peak age of paediatric adenoma was between 15 and 18 years, and macroadenomas comprised 75% of the cases, which is consistent with the results reported by Tetsuro *et al*^10^ The majority of paediatric pituitary adenomas were secreting adenomas and accounted for 73.2% of the cases in our series, which is consistent with prior reports.^5^ ^10^ ^11^ The higher incidence of secreting adenomas in paediatric patients may be associated with the powerful function of the pituitary gland in adolescence.

In this study, the rate of TR was 87.5% (table 2), which is comparable to previously reported rates of 77.8%^12^ and 81.5%.^13^ In our clinical experience, the anatomical features of the nasal and sphenoid sinuses in adolescent patients, including narrow nares and incomplete pneumatisation of the sphenoid sinus, did not affect the removal of tumours via endoscopic endonasal transsphenoidal approach (EETA) in this series. According to anatomical research results, the sphenoid sinus is well pneumatised in patients >10 years of age,^3^ and sphenoidal septations in paediatric patients older than 14 years are similar to those of adults.^2^ Additionally, the nares in some patients with GH-secreting tumours were wider than the nares of adults. In the present study, the peak age of patients was 15–18 years (91.1%), and most patients >15 years old underwent normal endoscopic procedures similar to those used in adult patients. For some patients whose sphenoid was incompletely pneumatised, imaging navigation was used to locate the sellar floor, and the tumour was accessed without difficulty.

In this study, 94.2% of patients experienced visual remission after surgery, which was consistent with a previously reported rate.^10^ We attributed this excellent result to effective decompression of the optic chiasm and nerves via the endoscopic approach and the surgeon's experience. Increased preoperative hormone levels may be associated with dysfunction or abnormalities of the pituitary gland resulting from tumour compression;^14^ resection of the tumour was crucial for relieving the hyperhormonal symptoms. For the treatment of prolactinomas, medical therapy should be preferred and was recommended in our institute. Surgery is chosen when patients encountered intolerable side effect of the drugs, or no response to medical therapy, or occurred pituitary apoplexy requiring emergent surgical intervention. Of all patients with secreting adenomas, 92.7% showed a reduction in hormone levels, and normalisation was achieved in some patients, which is similar to the proportion previously reported in the adult population.^15^

CSF leakage is one of the most common complications of EETA, and the reported rate in adults ranges from 1.3% to 15%.^16–23^ In paediatric patients, the rate ranges from 8% to 13%.^24–27^ Many techniques and materials have been used to repair defects of the skull base to prevent CSF leakage related to the endoscopic transsphenoidal approach.^28^ ^29^ Introduction of a vascularised PNSF for reconstruction of the skull base by Hadad *et al*^30^ greatly decreased the rate of CSF leakage, and subsequently, this technique began to be used by many centres.^31^ ^32^ In our study, the CSF leakage rate was 3.5% (table 3), which is lower than previously reported rates in paediatric patients.^24–27^ The occurrence of CSF leakage may be associated with surgical repair techniques, aggressiveness of the resection, location of the tumour and tumour adherence to surrounding neurovascular structures.^25^ Of the factors affecting CSF leakage following transsphenoidal surgery, the surgeon's technique of reconstruction of the skull base is thought to be the most important. The unparalleled detailed magnification of the surgical field provided by an endoscope permits the surgeon to visualise a tiny fistula and repair it. We typically applied autologous fat or fascia lata as well as synthetic dura to repair dural defects and used PNSF when intraoperative CSF leakage was suspected or confirmed, as described in the literature.^2^

Postsurgical DI is a neurogenic form of injury to the magnocellular neurons in the hypothalamus, where arginine vasopressin is produced and transported to the posterior pituitary gland via the hypothalamo-hypophyseal tract. Many factors, such as the tumour size, adherence to surrounding structures, histopathology and surgical technique, can result in DI. Care should be taken to preserve neurovascular structures and minimise injury to critical structures, including the hypothalamus, infundibulum and the neurohypophysis, during the surgical approach.^33^ In the present study, transient DI occurred in five (8.9%) patients (table 3), and one (1.7%) patient had persistent DI, which was treated with hormone replacement. These results are comparable to reported transient and persistent DI rates.^34^ ^35^

Hypopituitarism, indicated by decreased levels of pituitary hormones, also commonly occurred in paediatric adenoma patients. Four (7.1%) patients developed hypopituitarism in this study (table 3), and the rate was comparable to previously reported rates in adults that range from 1.4% to 19.8%.^4^ ^15^ ^16^ ^18–20^ The reason may be associated with the postoperative destruction or injury of the pituitary gland, pituitary stalk damage and dysfunction of the anterior pituitary; hormone replacement therapy was commonly required.^10^

## Conclusion

EETA is a safe and effective surgical option and can be performed to resect paediatric pituitary adenomas with outcomes comparable to those of adult patients.
